# Systemic thrombolysis and endovascular thrombectomy in severe acute ischemic stroke after dabigatran reversal with idarucizumab

**DOI:** 10.1002/ccr3.1446

**Published:** 2018-02-27

**Authors:** Quentin Binet, Frank D. Hammer, Olivia Rocrelle, André Peeters, Christophe Scavée, Cedric Hermans

**Affiliations:** ^1^ Hemostasis and Thrombosis Unit Division of Adult Hematology Cliniques Universitaires Saint‐Luc Brussels 1200 Belgium; ^2^ Division of Radiology Cliniques Universitaires Saint‐Luc Brussels 1200 Belgium; ^3^ Division of Neurology Cliniques Universitaires Saint‐Luc Brussels 1200 Belgium; ^4^ Division of Cardiology Cliniques Universitaires Saint‐Luc Brussels 1200 Belgium

**Keywords:** Acute ischemic stroke, dabigatran etexilate, idarucizumab, thrombectomy, thrombolysis

## Abstract

Patients presenting with an acute ischemic stroke despite dabigatran therapy (last intake <24 h or unknown) should be evaluated for reversal by idarucizumab, making them eligible for safe and effective intravenous thrombolysis. It has been shown to be feasible, well‐tolerated, and easy to manage in an emergency room or stroke unit.

## Introduction

Dabigatran etexilate (Pradaxa^®^; Boehringer Ingelheim Pharma GmbH & Co. KG, Germany) is an oral direct thrombin inhibitor. In contrast to vitamin K antagonists and just like the other direct oral anticoagulants (DOAC), it has decreased rates of hemorrhagic complications, does not require monitoring, and has minimal dietary or drug interactions. Common indications include deep venous thrombosis, pulmonary embolism, and the prevention of embolic complications in patients with nonvalvular atrial fibrillation (NVAF).

Patients presenting with an acute ischemic stroke despite dabigatran therapy, cannot benefit from intravenous thrombolysis (IVT) because of the anticipated increased risk of hemorrhagic transformation. Several case reports 1, 2 indicated that idarucizumab (Praxbind^®^; Boehringer Ingelheim Pharma GmbH & Co. KG, Germany) – a humanized monoclonal antibody fragment designed for rapid antagonization of the anticoagulant effects of dabigatran – may reduce the risk of symptomatic intracranial hemorrhage (sICH) and should be considered for acute stroke patients arriving in the IVT time window. Hereunder, we report a well‐documented case of successful systemic thrombolysis and thrombectomy after anticoagulation reversal for an acute ischemic stroke in a patient treated by dabigatran etexilate.

## Case Report

A 55‐year‐old woman was brought by ambulance to our emergency department for sudden onset of severe headache, slurred speech, and conjugate eye deviation.

Her medical history consisted in hypertension under tritherapy (bisoprolol 5 mg od, furosemide 20 mg od, and spironolactone 25 mg od) and paroxystic atrial fibrillation – with a known intra‐auricular thrombus and multiple systemic embolisms – treated by dabigatran etexilate 150 mg bid (last intake <2 h ago). She stopped smoking 18 years ago, but accounts for 30 pack‐years.

Neurological status on admission was NIHSS (National Institutes of Health Stroke Scale) 20, with findings of arousal on stimulation, mutism, right facial paralysis on Marie–Foix maneuver, partial gaze palsy correcting with oculocephalic reflex, right arm plegia, severe right leg paresis, and complete right arm sensory loss. Babinski's sign was positive on the right.

**Figure 1:**
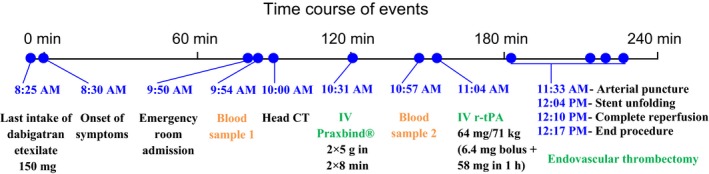
Time course of events.

**Figure 2:**
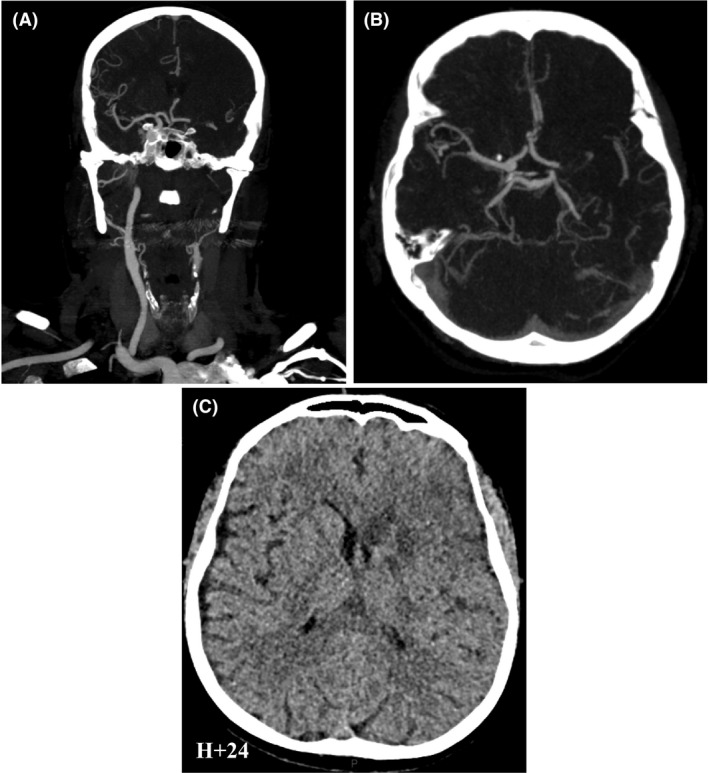
Cerebral imaging. (A) Head and neck CT angiography in acute setting showed an absence of opacification of the cervical segment of the left internal carotid artery. (B) Head CT angiography in acute setting revealed a hyperacute middle cerebral artery occlusion. (C) Follow‐up noncontrast CT on day 1 showed a left lenticulostriate infarct without hemorrhagic transformation.

A time course of the patient's management is found in Figure 1. Initial additional tests included (1) an electrocardiogram indicating atrial fibrillation, (2) a blood sample showing disturbances in hemostasis testing (an activated partial thromboplastin time (aPTT) of 37.4 sec, an international normalized ratio (INR) of 1.18 and a thrombin time of 80.7 sec) – as expected in a patient treated by dabigatran (therapeutic serum level of 61.4 ng/mL) –,and (3) a head computed tomography angiography (CTA) highlighting an hyperacute left carotid T occlusion (that is the occlusion of the intracranial portion of the internal carotid artery, extending into the middle and anterior cerebral artery) (Fig. 2A and B).

Because the time window was favorable for an IVT, we administered 2 × 5 g of idarucizumab intravenously and performed a blood puncture 10 min later, showing a massive decrease in dabigatran serum levels (0.9 ng/mL) and normalization of the aPTT (27.5 sec) and thrombin time (14.2 sec). (Table 1) Given the patient's weight of 71 kg, we administered 64 mg of recombinant tissue plasminogen activator (r‐tPA) intravenously.

**Table 1:** Hemostasis testing before (1) and after (2) idarucizumab administration. Abnormal values are written in italic letters

	Blood sample 1 (9:54 am)	Blood sample 2 (10:57 am)	Reference values and units
Activated Partial Thromboplastin Time (aPTT)	*37.4*	27.5	25.1–36.5 sec
International Normalized Ratio (INR)	1.18	1.14	0.80–1.20
Prothrombin time	13.80	13.30	9.35–14.30 sec
Thrombin time	*80.7*	14.2	10.0–18.0 sec
Fibrinogen	*564*	*538*	150–450 mg/dL
D‐dimers	<250	355	<500 ng/mL
Dabigatran	61.4	0.9	ng/mL

Hereafter, we performed an urgent mechanical thrombectomy: single pass with a 6 × 30 mm stentriever (Solitaire, Medtronic), deployed into the M1 segment of the middle cerebral artery and the distal internal carotid artery, and retrieved under suction through a balloon guiding catheter (8 Fr Cello, Medtronic). A thrombus of probable embolic origin was removed and complete reperfusion was obtained no later than 225 min after symptom‐onset.

The patient was hospitalized in the stroke unit for further observation and investigation. The control head CT at 24 h revealed an infarcted left lenticulostriate region, without stigmas of hemorrhagic transformation (Fig. 2C). A transthoracic and transesophageal echocardiography were performed and revealed a biauricular dilatation with a thrombus formation in the left auricle. Evolution was marked by a quick but partial recovery. The patient was discharged on day 8 with a NIHSS of 5 (persistent aphasia and hypoesthesia of both right limbs) and a modified Rankin scale at 3 (moderate disability: requiring some help, but able to walk without assistance). Revalidation was continued at home, consisting in neuropsychological therapy, speech therapy, occupational therapy, and physiotherapy.

## Discussion

Theoretically, on the basis of the daily dosing regimen for NVAF, treatment with IVT could be considered when it is started 12 h after the last dose of dabigatran in patients with normal renal function and no concomitant use of P‐glycoprotein and cytochrome P450 inhibitors 3. In practice, however, IVT is discouraged in acute stroke patients receiving their last DOAC dose <24–48 h or less than two halftimes ago (longer times are consistent with a very low probability of clinically relevant anticoagulant effect) 4. But the rapid reversal of the anticoagulant effect before IVT may be a new strategy. Until now, dabigatran is the only DOAC with a specific and approved antidote for emergency use: idarucizumab, which is a humanized monoclonal antibody fragment that binds dabigatran and irreversibly neutralizes the anticoagulant effect. The reversal effect has been shown to be quick (minutes) and sustained (12 h) 5. Indeed, the blood analysis performed 10 min after idarucizumab perfusion in our patient showed a rapid decrease in dabigatran serum concentration and a normalization of clotting parameters, in particular of the thrombin time (Table 1). If the stroke is related to a large vessel occlusion, endovascular thrombectomy may be considered as an alternative, or – as in our patient – as a complementary treatment option.

To the best of our knowledge, this is the only reported case of dabigatran reversal before IVT that has a good outcome despite a baseline NIHSS of 20. Previously published cases usually had good outcomes as well, but were generally of lower initial severity 1, 2. A systematic review of 21 patients with a median baseline NIHSS of 10 by Pikija et al. 1 identified 16% of unfavorable outcome (increase in NIHSS, recurrent stroke, symptomatic post‐thrombolysis intracranial hemorrhage, or death). No systemic bleeding, venous thrombosis, or allergic reactions were reported. However, this could be the reflection of a reporting and publication bias. Indeed, studies with positive results have usually a greater chance of being written and published than those with negative results. Although idarucizumab has shown clear efficacy in reversing dabigatran‐induced coagulopathy, its overall effects on patient outcome have not been proven 6. This case report aims, with the clinical context and accurate laboratory tests of dabigatran plasma level during reversal, at a better selection and follow‐up of patients who may benefit from idarucizumab. No causal links can be drawn from single case reports or series, and further prospective studies are warranted to evaluate the optimal management strategy.

Guidelines suggest reinstitution of anticoagulation after 1 day in patients with transient ischemic attacks (TIA) and after 3, 6, or 12 days for respectively minor (NIHSS < 8), moderate (NIHSS 8–16), or severe (NIHSS > 16) strokes 7. Hemorrhagic transformation needs to be excluded prior to treatment initiation. In our patient, however, despite the severity of initial presentation (NIHSS = 20), the intake of dabigatran 150 mg bid was resumed on the third day after the stroke given the high embolic risk of the known thrombus in the left auricle. There has been no hemorrhagic complication.

## Conclusion

This case report, along with previous reports, adds to the evidence that every patient presenting with an acute stroke despite dabigatran therapy (last intake <24 h or unknown) should be evaluated for reversal by idarucizumab, making him eligible for safe and effective IVT. It has been shown to be feasible, well‐tolerated, and easy to manage in an emergency room or stroke unit. Moreover, the availability of a specific reversal agent is an important factor to take into consideration when choosing an anticoagulant in clinical practice.

## Authorship

QB: wrote the paper. FDH: performed endovascular thrombectomy. OR, AP, CS, and CH: contributed to the medical management of the patient. All the authors critically reviewed and approved the final version of the paper.

## Conflict of Interest

The authors declare that there is no conflict of interest regarding the publication of this paper.

## References

[1] Pikija, S. , L. K. Sztriha , J. Sebastian Mutzenbach , S. M. Golaszewski , and J. Sellner . 2017 Idarucizumab in dabigatran‐treated patients with acute ischemic stroke receiving alteplase: a systematic review of the available evidence. CNS Drugs 31:747–757. 28808918 10.1007/s40263-017-0460-x PMC5573762

[2] Kermer, P. , C. C. Eschenfelder , H. C. Diener , M. Grond , Y. Abdalla , K. Althaus , et al. 2017 Antagonizing dabigatran by idarucizumab in cases of ischemic stroke or intracranial hemorrhage in Germany ‐ A national case collection. Int. J. Stroke 12:383–391. 28494694 10.1177/1747493017701944

[3] Cappellari, M. , and P. Bovi . 2015 Intravenous thrombolysis for stroke in patients taking non‐VKA oral anticoagulants: an update. Thromb. Haemost. 114:440–444. 25809649 10.1160/TH14-11-0973

[4] Heidbuchel, H. , P. Verhamme , M. Alings , M. Antz , H. C. Diener , W. Hacke , et al. 2016 Updated European Heart Rhythm Association practical guide on the use of non‐vitamin‐K antagonist anticoagulants in patients with non‐valvular atrial fibrillation: executive summary. Eur. Heart J. 38:2137–2149. 10.1093/eurheartj/ehw058 PMC5837231 27282612

[5] Eikelboom, J. W. , D. J. Quinlan , J. van Ryn , and J. I. Weitz . 2015 Idarucizumab: the antidote for reversal of dabigatran. Circulation 132:2412–2422. 26700008 10.1161/CIRCULATIONAHA.115.019628

[6] Vornicu, O. , A. S. Larock , A. S. Dincq , J. Douxfils , J. M. Dogné , F. Mullier , et al. 2017 Idarucizumab for the treatment of hemorrhage and dabigatran reversal in patients requiring urgent surgery or procedures. Expert. Opin. Biol. Ther. 17:1275–1296. 28728489 10.1080/14712598.2017.1349749

[7] Hankey, G. J. , B. Norrving , W. Hacke , and T. Steiner . 2014 Management of acute stroke in patients taking novel oral anticoagulants. Int. J. Stroke 9:627–632. 24891030 10.1111/ijs.12295 PMC4149783

